# Chip-encoded high-security classical optical key distribution

**DOI:** 10.1515/nanoph-2024-0188

**Published:** 2024-06-13

**Authors:** Bo Wu, Hailong Zhou, Jianji Dong, Yinfang Chen, Ninghua Zhu, Xinliang Zhang

**Affiliations:** Wuhan National Laboratory for Optoelectronics, School of Optical and Electronic Information, 12443 Huazhong University of Science and Technology, Wuhan 430074, China; Optics Valley Laboratory, Wuhan 430074, China; State Key Laboratory of Integrated Optoelectronics, Institute of Semiconductors, Chinese Academy of Sciences, Beijing 100083, China; Xidian University, Xi’an, China

**Keywords:** optical key distribution, reciprocity, incoherent matrix, silicon photonics

## Abstract

The information security plays a significant role in both our daily life and national security. As the traditional algorithm-based secure key distribution (SKD) is challenged by the quantum computers, the optical physical-layer SKD has attracted great attentions such as quantum SKD, chaos SKD, and reciprocity-based SKD. However, the cost of quantum SKD is still unaffordable and the latter two classical SKDs are only reliable with some preshared information or under simple eavesdrop. So far, there still lacks a high-security and low-cost optical SKD scheme. In this paper, we propose and demonstrate a high-security chip-encoded classical optical SKD paradigm based on the reciprocity of incoherent matrix. The security of SKD is facilitated by the incoherence of input light, and it is the first time that the classical optical SKD is achieved with silicon photonic chips and commercial optical fiber link. Experimentally, we set up a chip-to-chip communication link and achieve key generation rate of 100 bit/s over a 40 km single mode fiber, with key error rate of only 1.89 %. Moreover, we demonstrate the key capacity expansion of the proposed scheme with four-channel wavelength division multiplexing. Our proposal paves the way for the low-cost, high-security, and miniaturized optical SKD.

## 1 Introduction

The secure key distribution (SKD) technology is the first step of secret communication and has become an indispensable part of the current information society [1], [2]. The traditional SKD relies on the algorithm-based protocols such as Diffie–Hellman key exchange, which takes an ordinary eavesdropper exponential time to crack. However, the advancement of quantum algorithm and prototype has exerted a big threat on the algorithm-based SKD protocols [3]. After that, the SKD protocols based on the rich physical property of optical system and commercial optical fiber communication link have emerged as more secure substitutes, including quantum SKD [4], [5], [6], [7], [8], [9], [10], chaos SKD [11], [12], [13], [14], [15], [16], reciprocity-based SKD [17], [18], [19], [20], [21], and so on. Quantum SKD is inherently impeccable because it takes advantages of the unclonable property of photons, a theorem of quantum mechanism. Although the key generation rate (KGR) of more than 100 Mbit/s has been reported with quantum SKD, the cost of single photon detector is still too high [22]. Chaos SKD leverages the randomness of the chaos source and the selective modes as the guarantee of security. But it necessitates a common chaos source shared between the users, which is a rigorous condition and will become unsafe when the source is obtained by the eavesdropper. Additionally, there are some other proposals based on ultralong fiber laser, whose characteristic such as free spectrum range (FSR) and lasing state can be collaboratively maintained by the shared key [23], [24] to ensure security. However, they still encounter a potential risk of signal injection attack [23], [24], [25]. The reciprocity-based SKD is one of the most promising techniques to achieve physical-layer key distribution because its security does not depend on any shared physical information in advance. The reciprocity of the transmission channel inherently offers the same key between the legitimate users. Currently, the KGR of more than 10 Gbit/s over transmission distance of 50 km have been realized [21]. However, under specific attack of coherent optical signal, the key information can still be deduced by the eavesdropper. An optical SKD method featuring both low cost and high security is still imperative. From the perspective of hardware implementation, the miniaturization and low energy consumption of SKD system are the inevitable demand of market. Silicon photonic platform perfectly meets these requirements and has been widely adopted in optical quantum SKD. Apart from the advantages of size and energy consumption, it has great potential of performing highly reconfigurable optical operations and has become the greenhouse for optical matrix computing [26], [27], [28]. Exploring these merits of silicon photonic chip, classical optical SKD is promising to exert its advantages of low cost to the maximum.

In this paper, we novelly utilize the reciprocity of incoherent transmission matrix to realize a high-security classical optical SKD. Unlike the coherent reciprocity, the eavesdropper cannot derive the correct information of the transmission link in the incoherent system, which makes the system immune to the known attack. We integrate the core part of the SKD system on a silicon photonic chip and accomplish the chip-to-chip classical optical SKD over a 40 km single mode fiber. To the best of our knowledge, it is the first chip-assisted classical optical SKD, which fully leverage the high integration density and strong reconfigurability of silicon photonics. The theoretical KGR of more than 100 Gbit/s and experimental KGR of 100 bit/s with key error rate (KER) of only 1.89 % are presented as a demonstration. In addition, the four-fold increase of key capacity is achieved with four-channel wavelength division multiplexing (WDM) to highlight the scalability of the prototype. The results indicate the great potential of our proposed scheme in the low-cost and high-security optical SKD.

## 2 Results

### 2.1 Principle of incoherent optical SKD

**Figure 1:**
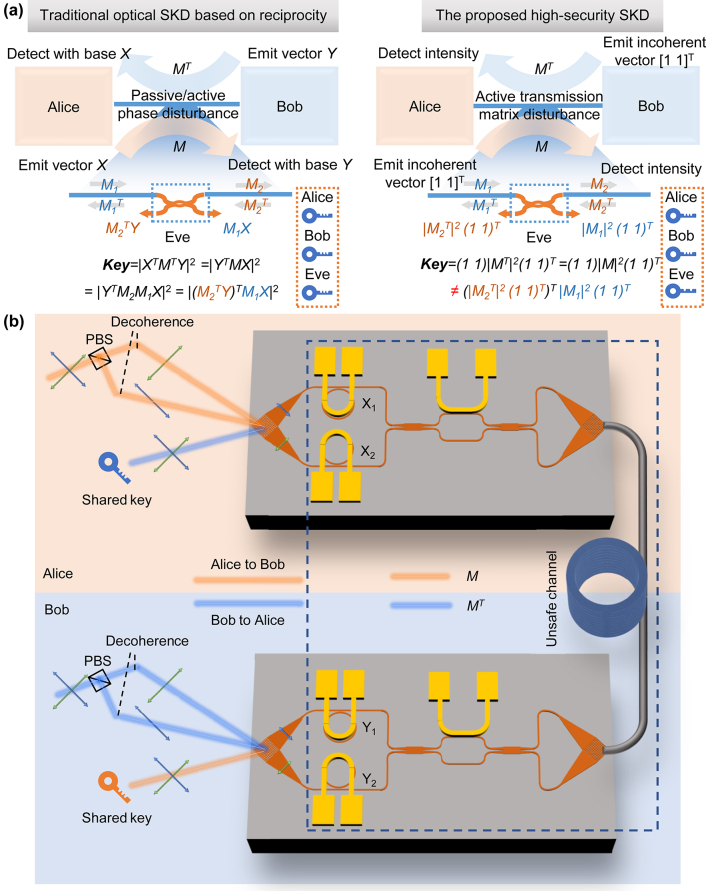
Working principle of the proposed optical SKD and its security analysis. (a) Comparison of the traditional optical SKD based on reciprocity and our proposed incoherent SKD. The Eve attacks by adding a 2 × 2 directional coupler and eavesdropping the corresponding vector. (b) The working flow of proposed SKD. Two decoherent orthogonally polarized beams are split into two waveguide channels and experience a linear transformation. In the other end, the light intensities of these two channels are summed up and become the shared key.

In this section, we recall the traditional reciprocity-based SKD and compare it with our proposed SKD to highlight our advantages of security. Both two SKDs rely on the reciprocity of linear optical system. For an *n*-input *n*-output linear optical system, the forward transmission matrix *M* of optical complex amplitude and backward transmission matrix *M* ^*T*^ of optical complex amplitude are mutually transposed [29]. The physical meaning of *M* depends on the base one chooses. For example, *M* is Jones matrix in the polarization-encoded channel [19], while *M* is mode-to-mode transmission matrix in the mode-encoded channel [30]. Regarding the traditional reciprocity-based SKD, the transmitters at two ends of communication channel emit a coherent optical vector *X*/*Y*, respectively, whose base can be either orthogonal optical port, mode, or polarization. At the receiver side, they detect the intensity of optical signal transmitted from the other end with base *X*/*Y*, which mathematically is an inner product. With the mapping relation shown in Figure 1(a) where the forward and backward transmission matrix are *M* and *M* ^*T*^, the shared key can be written as [20]:

(1) $$\text{Key}=|X^{T}M^{T}Y{|}^{2}=|Y^{T}MX{|}^{2}.$$

Here, |*X*|^2^ denotes a matrix constituted by the modulus square of each element of a matrix *X*. Since the legal users obtain the key information with their private base vector and communication channel between them at a certain time is always reciprocal regardless of the concrete value of *M*, they do not need any preshared information as other classical protocols do. To obtain the dynamic key, *M* is varying with time by adding passive asymmetric interferometer [18], [19] or active modulation [20]. Here, we propose a method to crack the key information. Assuming the eavesdropper is at somewhere of the fiber transmission link where the *M* can be divided into two parts: *M* = *M*_2_ *M*_1_, the key can be expanded as |(*M*_2_^T^ *Y*)^*T*^ *M*_1_ *X*|^2^. If the eavesdropper can derive the *M*_2_^T^ *Y* and *M*_1_ *X* simultaneously, the key will be cracked. Unfortunately, by adding a 2 × 2 directional coupler at where it is, *M*_2_^T^ *Y* and *M*_1_ *X* will be detected by the eavesdropper at the same time. For example, one can fabricate a silicon photonic chip consisting of polarization splitter rotator, directional coupler and 90° hybrid to crack the polarization-encoded SKD (see Supplementary Information S1 for the detailed deduction). To mitigate the potential insecurity, we propose a novel design of SKD, as shown in the right hand of Figure 1(a). Here, the emitted vector is an incoherent vector [1 1]^*T*^ encoded on the intensity of two orthogonally polarized light (see Supplementary Information S2 for the analysis of the optical incoherence). After transmitting through the actively modulated channel, the total light intensity is detected as shared key. Similarly, the shared key can be written as:

(2) $$\text{Key}=\left(\begin{array}{cc} 1 & 1 \end{array}\right)|M^{T}{|}^{2}\left(\begin{array}{c} 1\\ 1 \end{array}\right)=\left(\begin{array}{cc} 1 & 1 \end{array}\right)|M{|}^{2}\left(\begin{array}{c} 1\\ 1 \end{array}\right).$$

If the eavesdropper adopts the same method to crack the information, it can only get |*M*_2_^*T*^|^2^(1 1)^*T*^ and |*M*_1_|^2^(1 1)^*T*^, whose inner product is not equal to (1 1)|*M*_2_ *M*_1_|^2^(1 1)^*T*^. Owing to the incoherence in our scheme, the eavesdropper cannot directly retrieve the complete information of the transmission matrix. From the above analysis, we can conclude that our proposed method ensures a high security of the SKD.

Practically, the reconfigurability of *M* is realized using thermally tuned silicon photonic chip, as shown in Figure 1(b). The decoherence of two polarization is accomplished off-chip with a long fiber. Then the two polarizations are separated by a two-dimensional grating coupler and pass through the amplitude modulating unit (i.e., microring resonator [MRR]), unitary transformation (i.e., Mach–Zehnder interferometer [MZI] and fiber link), and another amplitude modulating unit. Finally, another two-dimensional grating coupler combines the two polarizations and the intensity coupled out is the final shared key. Taking the MZIs and transmission link as a unitary matrix *U*, the forward transmission matrix can be written as:

(3) $$M=\left(\begin{array}{cc} Y_{1} & 0\\ 0 & Y_{2} \end{array}\right)U\left(\begin{array}{cc} X_{1} & 0\\ 0 & X_{2} \end{array}\right),$$

where *X*_1/2_ and *Y*_1/2_ are the transmission coefficient of MRRs. The whole system is integrated on chip except for the incoherent source preparation and connected fiber link. Essentially, it should be noted that the chip architecture shown in Figure 1(b) is just a simple instance and there is a strong flexibility to design the SKD chip as long as it is linear and reciprocal, according to the working principle. Apart from that, in order to enhance the key capacity, more than one wavelength channel can serve as key in our proposed architecture. By combining more reconfigurable wavelength-selective devices such as MRR and asymmetric MZI with the wavelength-insensitive MZI mesh, different wavelength channels will have different transmission matrix and share parallel keys as a result.

### 2.2 Experimental demonstration

**Figure 2:**
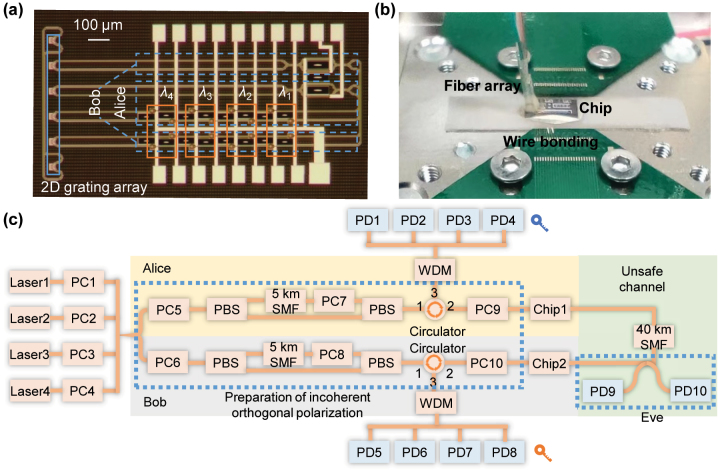
Chip preparation and the experimental setup. (a) The microscope image of the SKD chip fabricated in silicon photonic foundry. (b) The optically and electrically packaged chip for the SKD demonstration. (c) Experimental setup of the proposed SKD, including the optical signal preparation, transmission link and eavesdropper. PC, polarization controller. PBS, polarization beam splitter. SMF, single mode fiber. PD, photodetector. WDM, wavelength division multiplexer.

**Figure 3:**
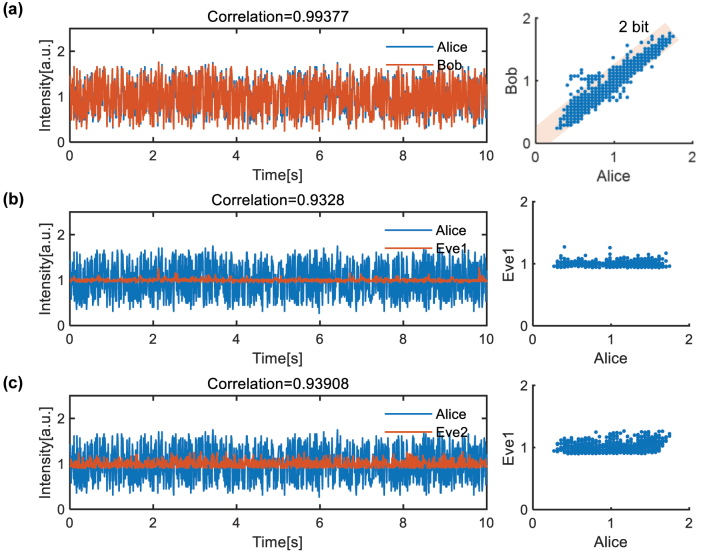
The experiment result of the SKD. The waveform received by (a) Alice and Bob, (b) Alice and Eve1, and (c) Alice and Eve2, which are both normalized to average intensity of one. Correlation between the shared key is represented as scatter diagram for these cases.

We fabricate the chip on the commercial 220 nm silicon on insulator (SOI) platform, as shown in Figure 2(a). All two-dimensional grating couplers are placed on a line to facilitate the optical package. The optical structures of Alice and Bob are fabricated on a single chip to make it convenient for test. Four MRRs are placed in every polarization route here to independently modulate four wavelength channels and achieve parallel SKD. The chip is optically and electrically well packaged with vertically coupled fiber array and wire bonding, as shown in Figure 2(b) (see Supplementary Information S3 for the characterization of the chip). To begin with, we demonstrate the effectiveness of one-channel SKD and its security. In this case, only one wavelength is involved and one of the four MRRs in the bus waveguide is working around resonance. The experiment setup is sketched in Figure 2(c) where only one laser is turned on in the present setup. The optical source of Alice and Bob is shared in our experiment for simplicity while they can also use their own independent source. Considering that Alice and Bob have independent optical source with different intensity, there will be a fixed multiple between their keys. Therefore, the two key sequences can always be normalized to have same average key intensity, which counteracts the influence of the fixed multiple. The blue dotted line accounts for the preparation of two decoherent orthogonal polarized light with same intensity. Optical fiber of 5 km is employed to produce the decoherence of original laser. The length of the fiber for decoherence can be freely chosen as long as the phase relation is fully scrambled. The on-chip optical structures of Alice and Bob are connected though a 40 km SMF to emulate the practical communication circumstance, and a 2 × 2 directional coupler is embedded inside the fiber link to emulate the eavesdropper. More details of the experiment can be found in the Methods. By fixing the random voltages on the MRRs and constantly modifying the voltages on the MZI, the waveform of Alice (PD1), Bob (PD5), Eve1 (PD9), and Eve2 (PD10) can be recorded in real time (the tuning mode is chosen under the guidance of numerical simulations in Supplementary Information S4). We modify the voltage with a time step of 10 ms, which is mainly restrained by the changing rate of multichannel voltage source. Here, we record 1,000 points and compare their waveform in Figure 3. All waveforms have been normalized to have average amplitude of one. The correlation between the two waveforms is defined as:

(4) $$\text{Corr}=\frac{\overset{⇀}{w_{1}}\cdot \overset{⇀}{w_{2}}}{\left|\overset{⇀}{w_{1}}\right|\left|\overset{⇀}{w_{2}}\right|},$$

where $\overset{⇀}{w_{1}}$ and $\overset{⇀}{w_{2}}$ are the vectors of the two derived waveforms. The correlation between Alice and Bob is 0.99377, while the correlation between Alice and Eve1/Eve2 is only 0.93280/0.93908. It should be noted that the waveform of Eve1/Eve2 is almost flat because only the unitary transmission matrix is varied. Although the correlation between legal users and eavesdropper is also numerically high, it has little influence on the fact that it has an ultra-high KER of 60.7 %/53.9 % for Eve1 and Eve2. Furthermore, we visualize the correlation by plotting the sampled points in the scatter diagram, from which we can identify a clear positive correlation between Alice and Bob while no correlation exists between Alice and Eve. The most points of Alice and Bob lie within the boundary of 2 bit, which means the shared key can be quantified as a 2-bit number. To further decrease KER, double threshold quantizer can be used (the rule of the quantizer can be found in Section 5). By setting an upper and lower bound of quantization level (±0.08), those easily confused data around quantization level can be removed and a better KER of 1.89 % can be attained with 476 effective data out of the original 1,000 data. Here, limited by the detection method, only direct light intensity eavesdropping is performed. However, from the theoretical analysis, we can deduce that our SKD system is highly safe regardless of any already-known attack method.

**Figure 4:**
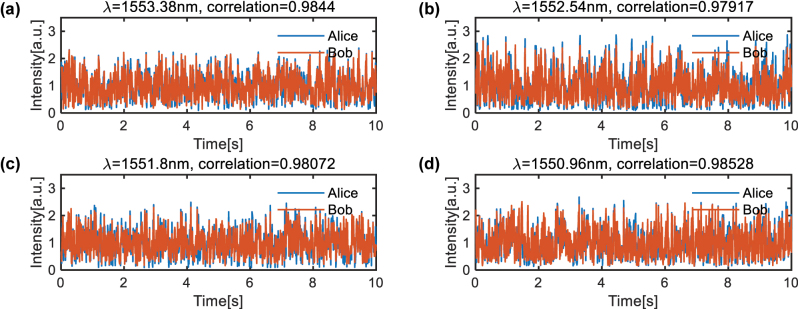
The experiment results of four-channel parallel SKD. Waveform of legal users with wavelength of (a) 1,553.38 nm, (b) 1,552.54 nm, (c) 1,551.8 nm, and (d) 1,550.96 nm.

To demonstrate the scalability, we perform a four-channel parallel SKD. Four wavelengths of 1,550.96 nm, 1,551.8 nm, 1,552.54 nm, and 1,553.38 nm are combined together and serve as the input laser source. At the end of the receiver, four wavelengths are separated with a demultiplexer and recorded by photodetectors, as shown by Figure 2(c). Four MRRs on the bus waveguide are precalibrated to resonate around the respective working wavelength and independently control the four channels. Figure 4 depicts the key waveform of four wavelength channels where the shared keys exhibit strong consistency in every channel. The slight degradation of correlation is mainly attributed to the wavelength dependence of Jones matrix of PC, PBS, and SMF, which indicates that the incoherent input optical vector of four wavelengths cannot be accurate [1 1]^*T*^ simultaneously. As a result, Eq. (2) will not hold. It can be compensated with wavelength-shaping on two polarization channels. Appling the same quantization method, we derive the KER of 3.85 %, 7.58 %, 4.24 %, and 5.04 % for the four channels under 2-bit accuracy, respectively. Meanwhile, 468, 462, 377, and 456 effective data are reserved with the low KER. The results provide a solid verification of the potential high key capacity of our proposed SKD system.

## 3 Discussion

KER of the SKD system can be improved with more precise polarization control, which is the main source of error in our experiment. And there are several factors related to the KGR of our system:

(I) In the device layer, although the rate of random source in our experiment is 100 bit/s, the state-of-art rate of DAC can be more than 100 Gsa/s (Keysight technologies). On the other hand, the thermal modulation limits the KGR to 20 kHz. To increase KGR, the modulation method can be altered from the thermal phase shifter to PN phase shifter with bandwidth of more than 100 GHz [31].
(II) In the algorithm layer, reciprocity of the channel should be relatively stable during the distribution of each key. That is, the point-to-point light propagation time (0.489 ms for 100 km single mode fiber) should be far less than the switch time of key. One way to avoid the restriction is to only modulate the chip state of one side for one key and apply the time division strategy (alternate key senders after certain period of time) so that the KGR can keep more than 100 Gbit/s within the time span, which will not influence the security of the system.
(III) In the physical layer, the stability of reciprocity can be maintained because the fiber link can be seen as an invariant during the distribution of one key. Although the KGR is also restricted by the linewidth of the optical source, it can be improved with broadband source input, as shown in the analysis in Supplementary Information S2.

Overall, we demonstrate, in different aspects, that the proposed SKD system is potential to achieve a high KGR. Apart from the speed limitation, only the simplest MRR and MZI unitary matrix are employed to modify the transmission matrix in our chip. For stronger security, more complex linear on-chip network such as singular value decomposition-based MZI mesh can be adopted [32]. Assisted by the high-speed carrier-depleted MRR and MZI modulator, a high-speed, multichannel SKD is promising to enhance the capacity of our SKD. Moreover, the incoherent paradigm of SKD is promising to be implemented on more optical dimensions as conventional reciprocal SKD, which makes it a generic SKD technique. Table 1 compares the key features of several optical SKD schemes. Among these schemes, our work exhibits both high security and low cost. The KGR of 100 bit/s also has great potential to reach the level of state-of-art with further improvement.

**Table 1:** Comparison of different optical key distribution schemes.

Scheme	Security	Cost	KGR
Quantum SKD [22]	High	High	100 Mbit/s
Chaos SKD [12]	Low	Low	10 Gbit/s
Fiber laser-based SKD [23]	Low	Low	100 bit/s
Reciprocity-based SKD [20]	Low	Low	4.3 Gbit/s
This work	High	Low	100 bit/s (potential 100 Gbit/s)

## 4 Conclusions

In conclusion, we propose a highly secure classical optical SKD based on the reciprocity of incoherent matrix. Compared with traditional classical optical SKD, the proposed one does not require any preshared information and is immune to most attack in the transmission link. Moreover, the classical optical SKD is first integrated on silicon photonic chip to fully unleash the potential of its reconfigurability and miniaturization. The chip-to-chip experimental demonstration of 100 bit/s SKD over a 40 km single mode fiber has been realized with low KER of 1.89 %. A four-channel WDM-based SKD further demonstrates the scalability of the proposed architecture. The scheme is low-cost, high-security and expected to play an important role in the further security-oriented communication.

## 5 Methods

### 5.1 Details of the experiment

In the experiment, we first tune PC1, PC2, PC3, and PC4 to align the polarization of four wavelength channels. Then, PC7 and PC8 are tuned to maximize the transmission of the following PBS. Next, we tune the PC5 and PC6 to balance the loss of two decoherent polarizations. Finally, the PC9 and PC10 are tuned to make the two-dimensional grating coupler split the correct polarization. The wavelength of laser is tuned to be slightly larger than the resonant wavelength of MRRs. The chip is thermally stabilized by a thermoelectric cooler (TEC). The optical signal output from the chip is separated from the input with an optical circulator. And the optical power is measured by a photodetector array electrically connected to the analog-to-digital converter (AD7606). The voltage applied to the heater is provided by the digital-to-analog converter (LTC2688). Both the AD7606 and the LTC2688 are controlled by a field programmable gate array chip (7K325T). The entire experimental system was controlled by a computer through serial ports.

### 5.2 The quantization method

In the main text, we adopt double threshold quantizer to quantify the received key signal [21]. Take the 2-bit quantization as an example. We first normalize the amplitude of detected signal to the span of 0–2. The quantization levels thus are 0.5, 1, and 1.5. The rule of double threshold quantizer can be written as:

(5) $$Q\left[n\right]=\left\{\begin{array}{c} 00,\text{if}\;S\left[n\right]<0.5-\Delta ,\\ 01,\text{if}\;0.5+\Delta <S\left[n\right]<1-\Delta ,\\ 10,\text{if}\;1+\Delta <S\left[n\right]<1.5-\Delta ,\\ 11,\text{if}\;S\left[n\right]>1.5+\Delta ,\\ \text{null},\text{else} \end{array}\right.$$

where *Q*[*n*] is the 2-bit quantization version of *S*[*n*]. The larger Δ is, the more points will be removed, which results in a lower KGR but a lower KER. In our experiment, when Δ is chosen to be 0.08, a KER of 1.89 % can be achieved in the cost of losing only half of the data.

## Supplementary Material

Supplementary Material Details
