# Supplementary material for: Chip-encoded high-security classical optical key distribution
Source: Nanophotonics. 2024 Jun 13;13(19):3717–25. doi: 10.1515/nanoph-2024-0188 (PMC11465989; doi:10.1515/nanoph-2024-0188)
Supplement: Supplementary file 1 — Supplementary Material Details [file j_nanoph-2024-0188_suppl_001.docx]

**Supplementary information for “****Chip-encoded high-security classical optical key distribution”**

Bo Wu^1^, Hailong Zhou^1, *^, Jianji Dong^1,2 *^, Yinfang Chen^3^, Ninghua Zhu^3^ and Xinliang Zhang^1,2,4^

*^1^ Wuhan National Laboratory for Optoelectronics, School of Optical and Electronic Information, Huazhong University of Science and Technology, Wuhan 430074, China*

*^2^ Optics Valley Laboratory, Wuhan 430074, China*

*^3^ State Key Laboratory of Integrated Optoelectronics, Institute of Semiconductors, Chinese Academy of Sciences, Beijing 100083, China*

*^4^ Xidian University, Xi’an, China*

^*^Corresponding author: [hailongzhou@hust.edu.cn](mailto:hailongzhou@hust.edu.cn); [jjdong@hust.edu.cn](mailto:jjdong@hust.edu.cn);

# S1. Cracking of the traditional reciprocity-based key distribution

The target of cracking is to derive the complex amplitude of detected optical signal (Fig. S1). When the optical signals of the two polarizations are split by two-dimensional grating coupler, their intensity |*E_x_*|^2^ and |*E_y_*|^2^ can be directly obtained. After that, they enter the module of 90° hybrid and generate four output signals carrying information [1]:

$$\begin{matrix} |E_{1}|^{2}=|E_{x}+E_{y}|^{2}=|E_{x}|^{2}|1+e_{y}|^{2}, \\ |E_{2}|^{2}=|E_{x}-E_{y}|^{2}=|E_{x}|^{2}|1-e_{y}|^{2}, \\ |E_{3}|^{2}=|E_{x}+iE_{y}|^{2}=|E_{x}|^{2}|1+ie_{y}|^{2}, \\ |E_{4}|^{2}=|E_{x}-iE_{y}|^{2}=|E_{x}|^{2}|1-ie_{y}|^{2}, \\ |E_{1}|^{2}-|E_{2}|^{2}=2|E_{x}|^{2}\mathrm{Re}(e_{y}), \\ |E_{4}|^{2}-|E_{3}|^{2}=2|E_{x}|^{2}\mathrm{Im}(e_{y}), \\ e_{y}=E_{y}/E_{x}. \end{matrix}$$

According to the relationship in Eq. (S1), *e_y_* can be fully determined and the complex amplitude of the two polarizations can be obtained. Therefore, the key information can be calculated as |(*M*_2_^T^*Y*)*^T^M*_1_*X*|^2^ by the eavesdropper.


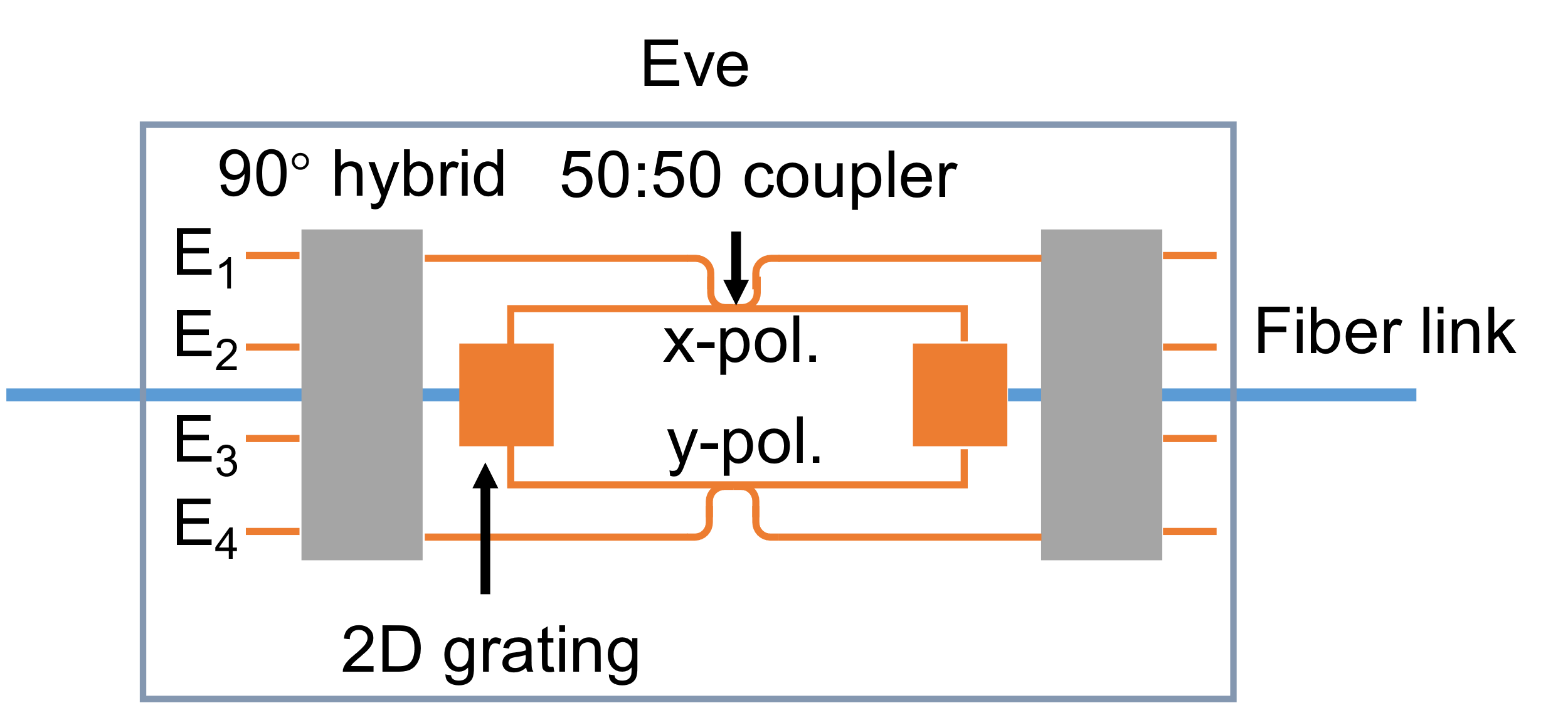


Fig. S1 The schematic diagram for cracking the key information of traditional reciprocity-based key distribution

# S2. Analysis of the optical incoherence

From another perspective, the input incoherent light can be regarded as a fast and randomly changing coherent vector [1 *e^iφ^*^(^*^t^*^)^]*^T^* where *φ*(*t*) is a fast changing phase term (The changing speed is determined by the bandwidth of optical source [2]). Therefore, the optical signal eavesdropper detect can be written as (Take the forward path *E_f_* as an example):

By assuming that the bandwidth of photodetector is much smaller than the changing speed of *φ*(*t*), the eavesdropper can only get a mean intensity of optical signal, that is, |*m*_11_|^2^+|*m*_12_|^2^ and |*m*_21_|^2^+|*m*_22_|^2^. Reversely, when the detection rate is faster than changing speed of *φ*(*t*), the security will be threatened. In the main text, owing to the narrowband feature of MRR, we only use a laser source of MHz bandwidth and long fiber to generate the incoherent light, which makes the changing speed of *φ*(*t*) to only MHz. Nevertheless, it is actually not hard to generate the incoherent light with THz changing speed of *φ*(*t*), which will make the SKD system impossible to crack with current detection system. As shown in Fig. S2(a), the two polarizations of broadband amplified spontaneous emission (ASE) source are decoherent and enter the spectrum partition module [3-5], which will partition the broadband spectrum into several channels with interval of 5 nm (0.625 THz). Next, the partitioned channels enter their individual MZI unit to modulate their intensity and are combined together, which are equivalent to MRRs in the experiment. Finally, the combined spectrum of the two polarizations enters the on-chip MZI meshes and the optical signals are emitted to fiber link through two-dimensional grating coupler. Figs. S2(b-d) shows three schemes to partition a broadband source. Alice and Bob can follow the same preparation setup to achieve the parallel SKD.


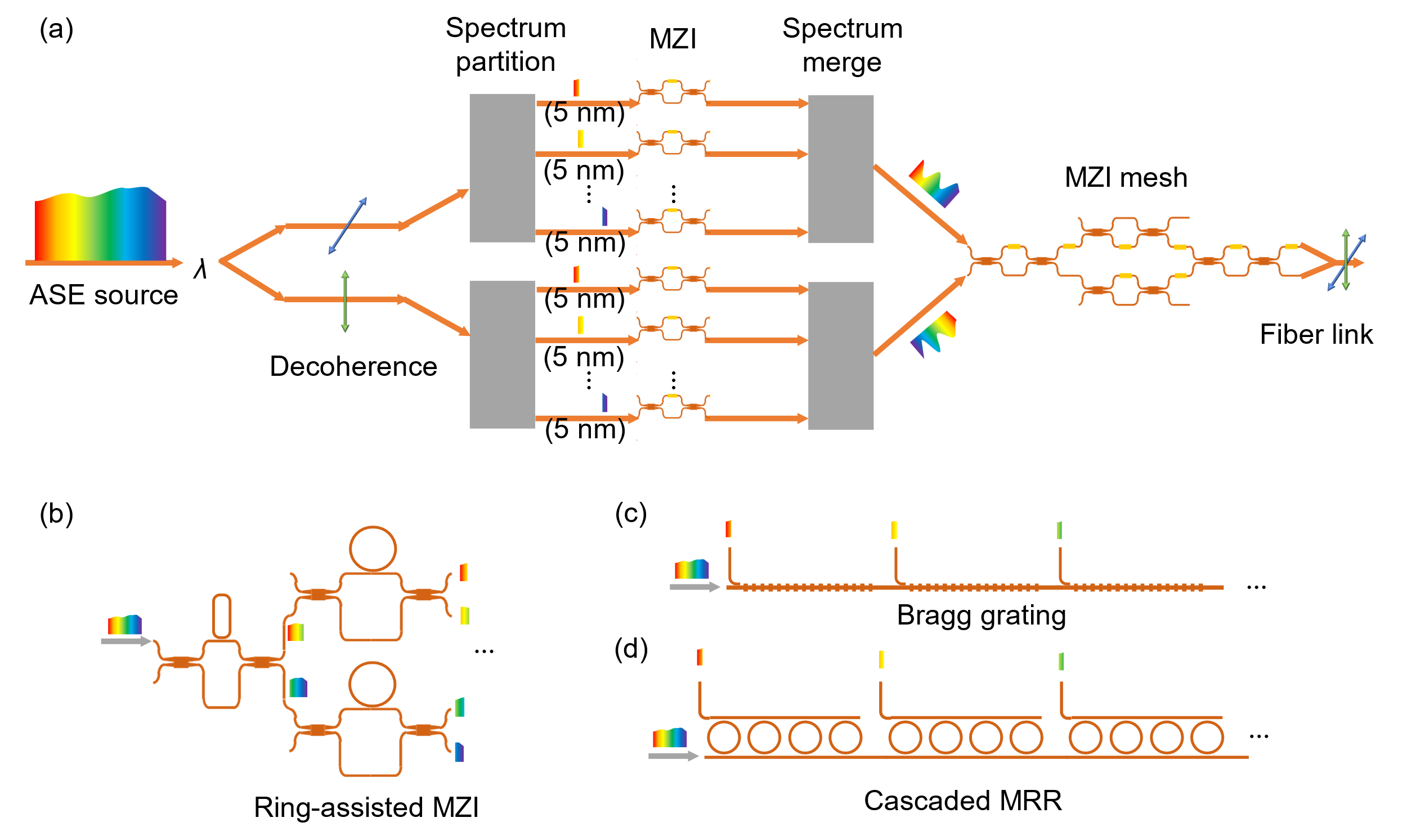


Fig. S2 (a) The prospect of broadband source-based optical key distribution with high security. (b) Wavelength interleaver based on ring-assisted MZI. (c) Wavelength partition based on Bragg grating [5]. (d) Wavelength partition based on cascaded MRRs [4].

# S3. Characterization of the SKD chip

Here we provide some basic features of the fabricated chip, as presented in Fig. S3. The two-dimensional grating coupler has a peak insertion loss of 6.53 dB and its 1 dB bandwidth is more than 30 nm. We further characterize the performance of MRR by carrying out an end-to-end test of the on-chip link, as shown in Fig. S3(b). MRR is all pass and with radius of 8 µm. The resonant wavelength of the each MRR is pre-tuned to the working wavelength. The notch depth of a single MRR is more than 10 dB. The MZI and MRR are modulated with TiN thermal heater whose π phase shift power consumption is about 18.4 mW and response time is about 7 µs.

Fig. S3 The basic character of the fabricated chip. (a) The transmission spectrum of two-dimensional grating coupler. (b) The transmission spectrum of calibrated on-chip MRRs.

# S4. Impact of different network architectures on the security of SKD

To explain the reason why we only tune the MZI unitary matrix in the experiment, we perform a simulation comparing the impact of different network architectures on the security of the proposed key distribution scheme. We run 10,000 rounds and plot the relationship of legal user and eavesdropper who performs the crack mentioned in the main text ((1 1) |*M*_2_|^2^|*M*_1_|^2^(1 1)*^T^*) in a scatter plot, as shown in Fig. S4. We can see a strong correlation when we tuned the SKD chip in the first case, which will significantly impair the security of the SKD. That’s why we only tune the MZI unitary matrix in the experiment (Fig. S4(b)). When the MZI mesh is universal, the MRRs can be tuned together with MZIs and shows a high security (Fig. S4(c)).


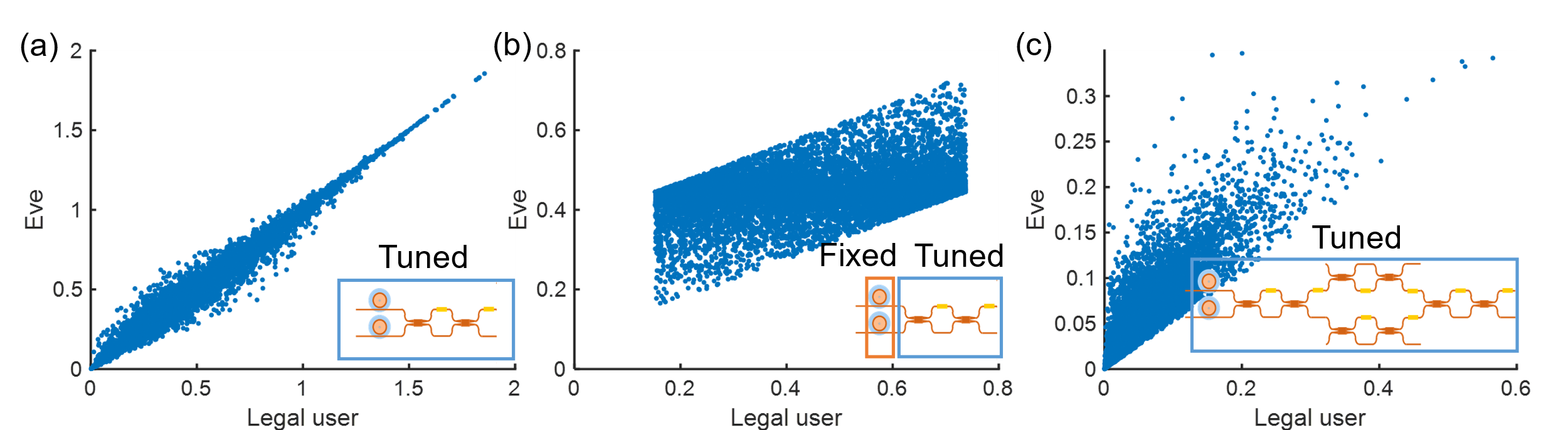


Fig. S4 The relationship between legal user and eavesdropper under the circumstance that (a) MZI mesh is unitary and both MRRs and MZIs are tuned. (b) MZI mesh is unitary and only MZIs are tuned. (c) MZI mesh is universal and both MRRs and MZIs are tuned.

**References**

1. Y. Wang, X. Li, Z. Jiang, L. Tong, W. Deng, X. Gao, X. Huang, H. Zhou, Y. Yu, L. Ye, X. Xiao, and X. Zhang, "Ultrahigh-speed graphene-based optical coherent receiver," Nature Communications **12**, 5076 (2021).

2. C. Huang, P. Y. Ma, E. C. Blow, P. Mittal, and P. R. Prucnal, "Accelerated secure key distribution based on localized and asymmetric fiber interferometers," Opt Express **27**, 32096-32110 (2019).

3. A. Rizzo, Q. Cheng, S. Daudlin, and K. Bergman, "Ultra-Broadband Interleaver for Extreme Wavelength Scaling in Silicon Photonic Links," IEEE Photonics Technology Letters **33**, 55-58 (2021).

4. D. Liu, J. He, Y. Xiang, Y. Xu, and D. Dai, "High-performance silicon photonic filters based on all-passive tenth-order adiabatic elliptical-microrings," APL Photonics **7** (2022).

5. D. Liu, J. He, M. Zhu, Y. Xiang, L. Zhang, M. Zhang, Y. Xu, and D. Dai, "High-Performance Silicon Photonic Filter Using Subwavelength-Structure Multimode Waveguide Gratings," Laser & Photonics Reviews **17**, 2300485 (2023).
